# Screening for Hepatocellular Carcinoma in Patients with Hepatitis B

**DOI:** 10.3390/v13071318

**Published:** 2021-07-08

**Authors:** Yashasavi Sachar, Mayur Brahmania, Renumathy Dhanasekaran, Stephen E. Congly

**Affiliations:** 1 London Health Sciences Center, Department of Medicine, Division of Gastroenterology, Western University, London, ON N6A 5A5, Canada; ysachar2023@meds.uwo.ca (Y.S.); mayur.brahmania@lhsc.on.ca (M.B.); 2 Centre for Quality, Innovation and Safety, Schulich School of Medicine & Dentistry, Western University, London, ON N6A 5W9, Canada; 3 Department of Medicine, Division of Gastroenterology and Hepatology, Stanford University, Stanford, CA 94305, USA; dhanaser@stanford.edu; 4 Department of Medicine, Division of Gastroenterology and Hepatology, Cumming School of Medicine, University of Calgary, Calgary, AB T2N 4Z6, Canada; 5 O’Brien Institute of Public Health, University of Calgary, Calgary, AB T2N 4Z6, Canada

**Keywords:** hepatitis B, hepatocellular carcinoma, cost-effectiveness, screening, risk stratification, alpha-fetoprotein, adherence, ultrasound

## Abstract

Chronic hepatitis B (CHB) infection is a significant risk factor for developing hepatocellular carcinoma (HCC). As HCC is associated with significant morbidity and mortality, screening patients with CHB at a high risk for HCC is recommended in an attempt to improve these outcomes. However, the screening recommendations on who to screen and how often are not uniform. Identifying patients at the highest risk of HCC would allow for the best use of health resources. In this review, we evaluate the literature on screening patients with CHB for HCC, strategies for optimizing adherence to screening, and potential risk stratification tools to identify patients with CHB at a high risk of developing HCC.

## 1. Introduction

Liver cancer is a leading cause of morbidity and mortality on a global scale, with approximately 30,000 deaths expected annually in the United States in 2020 [1], and significantly increased incidence rates modelled by 2030 world-wide [2]. The most common form of liver cancer is hepatocellular carcinoma (HCC), which accounts for approximately 75–85% of liver cancer cases [3]. HCC is within the seven most common cancers worldwide, and the third largest cause of cancer-induced mortality [4]. The leading cause of HCC worldwide is chronic hepatitis B virus (CHB) infection, which affects more than 250 million people world-wide [5]. Although every patient with CHB is at an elevated risk of developing HCC, this does not mean that each patient is at an equivalent risk. As demonstrated by the COVID-19 pandemic, surveillance programs can be easily disrupted. However, effective models of risk stratification can ensure that remaining resources are optimally allocated to the prioritization and prevention for those at the highest risk until the system returns to full capacity [6]. Several scoring systems have been created in an attempt to optimize the distribution of patients into different risk profiles, based on patient characteristics, along with viral characteristics [7]. The heterogeneity of the global CHB induced HCC population has made it difficult to develop a unified risk-scoring system, with differences among methodologies in terms of how these scoring systems identify, score, and evaluate relevant variables. This review aims to provide a comprehensive summary for the clinician, regarding screening for HCC in patients with CHB. Topics covered include reviewing the epidemiology of HCC in CHB, the state of research regarding screening patients with CHB for HCC, a summary of current guidelines for screening, highlighting the key literature regarding cost-effectiveness of screening, identifying barriers to adherence, discussing strategies to improve screening, and providing a complete and comprehensive analysis of existing HCC risk stratification models in patients with HBV.

## 2. Epidemiology of Liver Cancer and HBV

Chronic hepatitis B (CHB) infection accounts for 44–55% of HCC diagnoses world-wide, and those infected are at a 20-fold increased risk when compared to a non-infected population [8]. Although the prevalence of HCC in patients with CHB varies between populations, rates as high as 23.2% have been previously reported in Asia-specific studies [9]. High prevalence CHB regions are often resource limited and have a low human development index [10]; some of the most endemic regions include the Amazon Basin, Africa, Central, and Southeast Asia [11]. The relationship between CHB and HCC is further exemplified by the elevated risk of developing HCC in endemic regions, with sub-Saharan Africa and Eastern Asia being responsible for >80% of HCC cases [11]. This may change as HBV immunization programs gain stability in these regions [12]; however, there are limitations to its use as a prophylactic strategy against vertical transmission [13]. Although it is not a traditionally endemic region, the CHB population has been increasing in recent years in North America [14], with a 2020 study estimating approximately 1.59 million people having CHB in the United States of America [15]. This increase is mainly driven by external factors, such as an influx of immigration from countries with a high CHB prevalence. The increase in CHB has been paralleled by an increase in HCC, which is now the fastest growing cancer in the United States in terms of incidence [1]. Furthermore, the proportion of HCC cases resulting from CHB has also increased, with one American study demonstrating that the proportion of CHB-induced HCC cases grew from 4% to 21% [16]. Therefore, as CHB and HCC become more common in western populations, the burden of disease has shifted from its traditional localized distribution to a global impact.

## 3. Screening for Hepatocellular Carcinoma

### 3.1. Rationale for Screening

The rationale for any screening strategy is to identify patients at earlier stages of disease where treatment has a higher likelihood of success, as well as to identify risk factors that increase the risk of developing the disease, as to allow for interventions [17]. Factors to consider in the evaluation of any screening strategy are whether it is a disease of concern, and whether the screening test is effective, reasonable in cost, and improves outcomes [17,18]. Screening for HCC clearly meets these criteria, given that HCC is a public health concern, leading to a significant loss of life years and quality of life [19].

Currently, an ultrasound with the possibility of adding alpha-fetoprotein are recommended as the screening strategies for HCC [20,21,22,23,24]. At this time, there are only three randomized clinical trials comparing HCC surveillance versus no surveillance, with conflicting results related to methodological challenges. The first trial studied patients from Shanghai that were employed in factories, schools or private enterprises, with CHB or chronic liver disease; they were allocated by cluster sampling into those who received AFP and an ultrasound every 6 months (n = 8109) and a control group (n = 9711) enrolled between 1992–1994 [25]. In screened patients, 38 patients with liver cancer were identified after 12,038 person-years, and in the control group 18 patients were diagnosed after 9573 person-years. Patients identified in the screening group had a 1-year survival of 88.1% and two-year survival of 77.5%, and HCC were found at an earlier stage than the control group; the control group had a 0% 1-year survival. Although this study estimated an average cost of $1500 USD per early-stage HCC diagnosis, the number of false positive screening results was not reported, and would likely have increased the procedural and administrative cost of executing this program.

A similar study was conducted through 1993–1995 in Shanghai, consisting of 18,816 patients aged 35–59 with chronic hepatitis B. Participants were randomly allocated to screening (9373) with alpha-fetoprotein and ultrasounds every 6 months, versus a control group (9443) [26]. The surveillance arm identified 86 cases with 32 deaths from HCC, and the control arm had 67 cases with 54 deaths, leading to a risk ratio of 0.63 (95% CI 0.41–0.98) for the screening arm. Notably, this trial has been criticized for a lack of information about each group’s baseline characteristics, and how the outcome of death from liver cancer was defined [27]. Furthermore, the cohort likely has significant overlap with the last study by Yang et al. [25], given the population characteristics and time frame of recruitment.

The third randomized trial focused on men aged 30–69 between 1989–1995 in Jiangsu Province, China, using alpha-fetoprotein (AFP) levels every 6 months (n = 3712, mean follow-up 61.9 months) with a threshold of 20 μg/L, versus a control group (n = 1869, mean follow-up 62.8 months) [28]. Adherence rates for obtaining AFP were low at about 30%. A significantly larger number of cases were identified in the screening arm vs. the control arm at early stages (BCLC Stage 0-A [29]), with early detection rates of 29.6% and 6.0%, respectively. The 5-year survival between the groups did not differ at 4%, and the risk ratio in the screening arm was not statistically significant (RR 0.83; 95% CI 0.68–1.03), which may be related to the limited antiviral therapy available at the time of the study. The study has been criticized for the unclear randomization and allocation concealment techniques that were used [27].

A meta-analysis from 2014 which included the second and third trial, as well as 18 observational studies examining the role of screening in patients with chronic liver disease, concluded that it was uncertain whether systematic surveillance improved patient survival, and highlighted that more data were required given the relatively few studies on the CHB population [27]. A second meta-analysis published by Singal et al. [30] looking at patients with cirrhosis, suggested that the use of ultrasound was associated with a higher probability of diagnosing cancers at an earlier stage (OR 2.08, 95% CI 1.80–2.37) and improved survival (OR 1.90; 95% CI 1.67–2.17). These differences remained significant when accounting for lead time bias; however, only three publications looked at HBV patients in particular, with no subgroup analysis performed.

More recent epidemiological studies have shown potential survival benefits of HCC surveillance. One Canadian trial examined outcomes in patients with viral hepatitis diagnosed with liver cancer, and the impact of ultrasound screening prior to their diagnosis [31]. The 5-year survival in patients receiving routine surveillance (at least an ultrasound annually) was 31.93% (95% CI 25.77–38.24%) versus 20.67% (95% CI 16.86–24.74%) in patients who were not screened. Moreover, surveillance was associated with a mortality risk of 0.76 (95% CI 0.64–0.91), which indirectly supports the role of screening. Similarly, in patients with all-cause cirrhosis, screening for HCC has been suggested to have potential survival benefits [32,33,34]; although, a case-control study of the US Veterans Affairs cohort did not show a survival difference between those screened with ultrasounds and those who were not [35]. Current recommendations from the US National Cancer Institute suggest that screening patients at an increased risk does not lead to a decrease in mortality from HCC, as shown by fair quality evidence that is based on lead and length time bias [36].

Ideally, a randomized clinical trial in an attempt to best answer this question would be useful; however, this is unlikely to happen, given ethical concerns about the harm associated with no surveillance, as patients and practitioners prefer surveillance [37]. Further, answers to the true benefit of screening through cohort/case-control trials focusing on the role of screening patients with CHB will be important, given the limited high-quality literature to date. One potential barrier to a universal consensus regarding screening is the variation in efficacy of screening between regions. This is due to internal population factors, such as the prevalence of CHB and risk of tumor development based on prognostic factors of disease severity, in addition to health care resources available in the region. For example, although a male cohort between the ages of 70–79 may be at the highest risk of developing HCC, Japanese, African, and Chinese populations are skewed as significantly younger [21]. These differences in epidemiology are mirrored by regional differences in post-diagnosis outcomes [38], dependent on factors, such as availability of therapeutics and patient health status. Consequently, any analysis regarding the impact of surveillance may be limited to regional applicability, and if an RCT is conducted in the future it would be best to involve multiple regions, in an effort to create a generalizable scope of study.

### 3.2. Current Society Guidelines for HCC Screening

Currently, it is recommended by all major hepatology international societies to screen for liver cancer in patients with CHB deemed to be high risk with the use of abdominal ultrasound every 6 months, with variable recommendations regarding the inclusion of AFP. Although these guidelines are largely similar, there are some minor differences, i.e., the use of AFP and when to screen patients from an African background. We summarize the current society guidelines in Table 1.

AFP is a glycoprotein produced primarily during the ontogenesis of the yolk sack and fetal liver, with a suppressed expression following birth. However, increased AFP production has been associated with reparative growth following liver damage [39], as well as oncogenesis, acting as a pro-proliferative protein involved in regulating apoptosis, growth, and angiogenesis [40]. Due to its association with abnormal hepatocyte proliferation, AFP elevations are well documented in both malignant liver disease [40] and nonmalignant liver diseases, such as acute hepatic failure [41] and CHB [42]. Unfortunately, the use of AFP as a screening tool remains unclear. A recent meta-analysis evaluated the effectiveness of screening with ultrasound versus using ultrasound and AFP in detecting early cancer [43]. Thirty-two studies were identified in this study (consisting of 13,367 patients). Ultrasound identified any stage of HCC with 84% sensitivity (95% CI 76–92%), while identifying early-stage HCC with 47% sensitivity (95% CI 33–61%). The addition of AFP to ultrasound increased the sensitivity to 63% (95% CI 48–75%) for early-stage HCC, and to 97% (95% CI 91–99%) for all stages of cancer. Subsequently, the addition of AFP reduced the specificity to 84% (95% CI 77–89%) from 92% (95% CI 85–96%). There were some limitations to this meta-analysis, as it had no studies that were looking at survival. Moreover, most studies only looked at HCC at any stage vs. early HCC, which may overestimate surveillance test performance. Furthermore, negative screening tests were not confirmed by another modality, which may lead to verification bias. Overall, it is critical to note that using AFP by itself is not recommended, as it has poor sensitivity and specificity [44].

#### 3.2.1. Asian Pacific Association for the Study of the Liver (APASL)

The 2017 APASL guidelines [24] recommend abdominal ultrasounds with AFP every 6 months for HCC screening in patients with CHB cirrhosis in Asian females > 50 years, Asian males > 40 years, patients of African background > 20 years, and in patients with a family history of HCC (no specific starting age recommended) (Table 1). The AFP threshold recommended is 200 ng/mL to obtain the optimal positive likelihood ratio.

#### 3.2.2. American Association for the Study of Liver Diseases (AASLD)

The 2018 AASLD guidelines [22,23] recommend abdominal ultrasounds every 6 months with or without AFP. The latter is a significant change from the 2010 guidelines [45], which explicitly recommended against AFP, due to concerns with false positives and subsequent testing. The AFP threshold recommended by the AASLD is 20 ng/mL. High risk groups that are recommended for HCC screening include patients with cirrhosis secondary to CHB with either Child–Pugh A or B; Child–Pugh C patients who are non-transplant candidates are excluded, as treatment would likely not be feasible. Other patients recommended for screening include Asian females > 50 years, Asian males > 40 years, patients of African background > 40 years, patients with a first-degree relative with a history of HCC (no specific starting age recommended), and patients co-infected with hepatitis delta (HDV) (no specific starting age recommended) (Table 1).

#### 3.2.3. European Association for the Study of the Liver (EASL)

The 2018 EASL guidelines [21] advocate screening high risk patients with abdominal ultrasounds every 6 months, and do not recommend using AFP routinely, due to concerns regarding false-positive results in the context of active liver inflammation. Patients recommended for screening include those with Child–Pugh A or B cirrhosis; Child–Pugh C cirrhosis awaiting liver transplantation; and patients with chronic hepatitis B determined to be at an intermediate or high risk of developing HCC, based on PAGE-B classes for those of Caucasian background (Table 1).

#### 3.2.4. Canadian Association for the Study of the Liver (CASL)

The 2019 CASL guidelines [20] recommend performing abdominal ultrasounds every 6 months for patients deemed to be at a high risk for developing HCC. This includes patients with CHB cirrhosis, Asian females > 50 years, Asian males > 40 years, patients of African background > 20 years, and patients with a family history of hepatocellular carcinoma starting at age 40, as well as patients with HIV co-infection > 40 years (Table 1).

#### 3.2.5. Guideline Based Approach to Co-Infection

All four guidelines discussed above highlight the potential impact of co-infection with hepatitis C virus (HCV), HDV or HIV as a risk factor for developing HCC, although each set of guidelines emphasizes a different viral co-infection. The data for the impact of co-infection on its development is strongest in HDV [46,47], with data for HCV [48] and HIV [49,50] being weaker. Although there may be an increased risk with co-infection, there is little evidence regarding how screening guidelines should be changed. This likely explains the lack of explicit comment throughout guidelines, regarding the impact of co-infection and HCC screening; this is an important area for further research. That being said, it is important to recognize that both patients with mono-infection and significant co-infection undergo the same HCC surveillance protocol under the previously mentioned guidelines, suggesting the primary driver for surveillance in this situation is the oncogenic potential of CHB.

### 3.3. Cost Effectiveness of Screening Strategies for HCC

A key component of any screening strategy is ensuring it offers good value for money; that is, the strategy is cost effective. The majority of studies evaluating the cost-effectiveness of screening consider patients with cirrhosis, with a minority of studies looking at hepatitis B being mostly focused on Asian populations [51]. In studies comparing screening using abdominal ultrasound +/− AFP with no screening [52,53,54,55,56], screening programs were cost effective in all but one study [55].

There are two recent studies evaluating the cost effectiveness of imaging-based screening, as compared to no screening at all. A Taiwanese based study evaluated the cost effectiveness of screening patients with CHB with ultrasounds every 6 months versus no ultrasounds [56]. The base case was a 50-year-old individual who was followed over a 25-year time horizon. Screening had a cost of $5912.37 USD for 13.78 years of life, as compared to $557.10 for 13.53 years of life in the unscreened arm, working out to an incremental cost effectiveness ratio of $20,856.25/year of life gained. Sangmala and colleagues expanded this analysis, and evaluated multiple screening strategies in a Thailand population of patients with CHB between the ages of 40–60 with a lifetime time horizon [52]. The strategies evaluated in this model included abdominal ultrasound, AFP and US, computed tomography (CT), and magnetic resonance imaging (MRI) at either a 6-monthly or 12-monthly frequency. Without any form of screening, only 6.24 quality adjusted life years (QALY) were gained. The use of ultrasound or ultrasound and AFP for HCC screening were found to be the most cost effective strategies. Notably, CT and MRI based strategies were not cost effective.

A recent analysis evaluating the cost effectiveness of screening compares abdominal ultrasound with or without AFP for HCC screening. This analysis is novel in its consideration of the potential harm of unnecessary tests, due to false positives [57], which is not considered in previous studies. The cohort studied were patients with compensated cirrhosis over a lifetime horizon. The most cost effective strategy for the cohort was using US with AFP, with a cost of $1,254,173.20 USD for 6.02 QALY; both using US and no surveillance in this cohort was more expensive and less effective than US with AFP. Modelling the impact of false positive testing in the CHB population is an important area to evaluate, until then, extrapolation from the available evidence is required.

## 4. Challenges with Screening Adherence

Adherence by patients to screening programs is a key indicator of success. Unfortunately, a major challenge with HCC surveillance programs is that of poor adherence rates. A study from Washington State looking at 1137 patients with cirrhosis highlights this challenge. Over a 2-year period, 33% of this group had at least one ultrasound (intermittent surveillance), and those with CHB cirrhosis were found to be more likely to undergo surveillance. Notably, only 2% of patients in this study underwent consistent surveillance, defined as having an ultrasound every 6 months [32]. A recent meta-analysis looking at screening for patients at a high risk of developing HCC identified 22 studies involving 19,511 patients, showing an overall adherence rate of 52% (95% CI 38–66%). Looking at the subset of studies of patients with CHB (4 trials, 2651 patients), the adherence rate was significantly lower (32%, 95% CI 13–51%). A subsequent meta-analysis focusing on patients with cirrhosis identified 29 studies (n = 118,779 patients) with a surveillance rate of 24% (95% CI 18.4–30.1); however, higher surveillance rates were observed when patients were seen by gastroenterologists and/or hepatologists [58].

**Figure 1:**
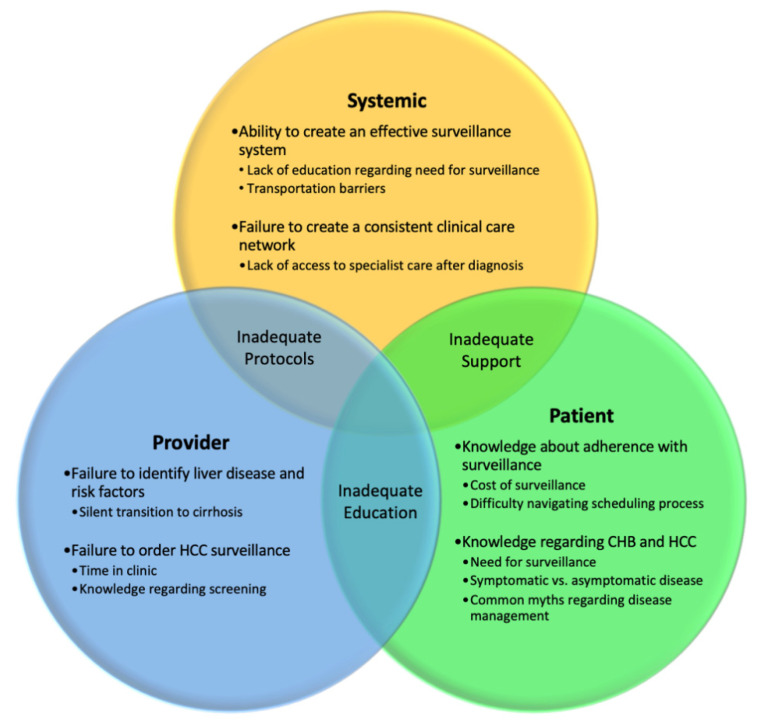
Factors Associated with Decreased Adherence to HCC Screening [37,59,65,66].

Screening programs are complex, and there are many reasons why lower adherence rates to screening are observed. Simply, there are system, provider, and patient-based factors, as outlined in Figure 1. Physician failure to order HCC surveillance was the most common reason for patients not receiving surveillance in two studies [59,60], with a separate investigation of physician surveillance practices finding only 22% relied on biannual imaging for HCC surveillance [61]. However, patient-driven non-adherence remains a significant determinant of surveillance inconsistency, with Singal et al. estimating patient non-adherence accounted for <10% of adherence failures [62] in their study. Patient factors can be further divided into those that are related to feasibility (i.e., cost, insurance coverage [62], difficulty navigating system, transportation [63]) and patient perceptions (i.e., knowledge regarding need for surveillance, HCC presentation, management [63]). Both patient and provider factors are further exacerbated by systemic flaws, with issues related to supporting surveillance infrastructure and clinical care network [64], identified by existing studies.

### Methods to Improve Adherence

A number of strategies have been studied in an attempt to improve adherence to HCC screening, including the education of primary care providers, nurse led clinics, mailed outreach, and radiology led programs [58]. Although all of these techniques have merit, the impact of these strategies are variable in efficacy. There have been four studies utilizing a post-intervention outcome of the proportion of patients meeting the gold-standard of having ultrasounds every 6 months. A randomized control trial evaluating outreach letters by mail showed that the rate increased from a baseline of 7% to 21% in the intervention arm [67]. Moreover, systematic changes may be associated with higher rates of surveillance, although likely coming at a higher cost. For example, in Australia, the introduction of a nurse-led clinic led to 53% of patients seen in the clinic having appropriate ultrasounds [68], while a more intense system redesign and patient education program led to 63% of patients being screened [69]. From the radiology provider side, a radiology recall program in the United Kingdom [70] led to 46% of patients achieving appropriate screening. There is no one-size solution, but likely a structured intervention whereby the payer, patient, provider, and system will be important to increase the adherence rate of screening.

## 5. Risk Stratification Systems for HCC Risk in Patients with HBV

When comparing the various models of risk assessment, there appears to be some common areas of contention, which are addressed differently by different models. Risk stratification scores for HCC risk in HBV have been previously reviewed [71]. Here, in Table 2 and Table 3, we present an updated critical summary of all published models of HCC risk assessment in patients with CHB. The majority of studies assessed in this review involve risk models, generated using homogenous populations. Although this is a result of the relatively homogenous populations in some of the countries in which these studies were generated (i.e., South Korea, Taiwan), this limits the utility of these models as international standards for HCC surveillance. In comparison, models generated in western countries, such as PAGE-B, have an advantage in terms of generalizability, as their study population for CHB often has a greater degree of ethnic diversity. However, as the number of models grows, newer studies such as the aMAP have been able to test their model in multiple populations of different ethnicities prior to publication, providing further support for the generalizability of their model. Although many studies report using gender as a risk factor, it is most likely that gender is incorrectly used, and this should be biological sex rather than the social concept of gender [72]; acknowledging this, we report the criteria as provided by the original publication. Ultimately, the majority of risk assessment models were reliant on follow-up studies, validating them in other population groups to strengthen their argument for generalizability.

Another area of difference between risk assessment models is the use of a three-tier vs. two-tier stratification of patient risk of developing HCC. While three-tier models are more likely to be statistically accurate than two-tier models, given they allow for a greater degree of objectivity over the data being analyzed, they are limited in their potential utility. Protocols in practice for patients with CHB [107] essentially culminate in a binary decision, and it must be determined whether the patient would be included in a screening protocol or not. Ultimately, three-tier models may have limited clinical applicability, as many of the systems in place for screening patients are not designed to have separate protocols for patients who are “high risk” vs. those categorized as “medium risk”. Thus, having a paralleled stratification of outcome and model is beneficial for ease of use, and mitigates confusion on how patients should be stratified into surveillance vs. screening.

A limitation of most existing risk scores is a lack of discrimination between the stages of fibrosis beyond a diagnosis of overt cirrhosis. Studies have demonstrated a similar 5-year HCC probability for patients with a histopathological diagnosis of cirrhosis and those with a diagnosis of advanced fibrosis [108], elucidating the need for a more nuanced approach to fibrosis when developing a risk score. An improved assessment of the degree of fibrosis may help improve the cost effectiveness of screening strategies; there are a number of non-invasive techniques for fibrosis evaluation, which have been reviewed in recent guidelines [109]. Further, antiviral-treated patients require accurate staging of fibrosis for updating their risk of developing HCC, as patients with an appropriate treatment response may present with a regression of fibrosis, a positive prognostic indicator for HCC risk [110].

A recent study suggested the utility of a model combining AFP and the FIB-4 model, to predict HCC outcomes for patients with compensated cirrhosis, due to CHB being treated with antiviral therapy [111]. However, the novel approach was not compared to existing HCC-CHB risk stratification models, and the reliability of FIB-4 for non-Asian patients with CHB has also been called into question [112]. To appropriately utilize a FIB-4-like system for CHB, further adaptation may be necessary, with novel modifications being capable of identifying fibrosis, and stratifying HCC risk in the CHB population currently being investigated [112,113].

## 6. Conclusions

Although screening for HCC in high-risk patients with CHB is considered the standard of care and has proven benefits, there remains significant gaps in the knowledge base. Outstanding questions include whether alpha-fetoprotein should be a part of the screening program, as well as the risk stratification of patients with CHB in order to tailor screening programs to those with the highest risk of HCC. From a practical standpoint, investigations on how to develop structured interventions involving the payer, patient, and system to improve adherence to screening programs will be needed to maximize the effectiveness of any of these programs. Ultimately, surveillance programs are necessary for the effective identification of early-stage cancer in high-risk patients. This paper identifies potential areas of improvement to further improve their impact and accuracy in a clinical setting.

## Figures and Tables

**Table 1:** Summary of International HCC Screening Recommendations.

^1^	APASL 2017	AASLD 2018	CASL 2019	EASL 2018
Screening Strategy				
Abdominal ultrasound (US)	Recommended every 6 months			
Alpha-fetoprotein (AFP)	Recommend AFP every 6 months with US	Can consider AFP every 6 months with US	AFP use not recommended	
**Patients for Which Screening is Recommended**				
Patients with cirrhosis	Yes	Child Pugh A/B	Yes	Child Pugh A/B
Asian men with chronic hepatitis B	>40 years old			PAGE-B ≥ 10
Asian women with chronic hepatitis B	>50 years old			
African men/women with chronic hepatitis B	>20	>40	>20	
Family history of hepatocellular carcinoma	Yes	Yes	Yes, >40 years old	No
Co-infected with hepatitis delta virus (HDV)	No	Yes	No	No
Co-infected with human immunodeficiency virus (HIV)	No	No	>40 years old	No

^1^ APASL: Asian Pacific Association for the Study of the Liver; AASLD: American Association for the Study of Liver Diseases; EASL: European Association for the Study of the Liver; CASL: Canadian Association for the Study of the Liver; PAGE-B: platelets, age, gender-HBV score.

**Table 2:** Critical Analysis of Untreated Patient Risk Assessment Models.

Name ^1^	Components	Strengths	Weaknesses
IPM [73]	Cirrhosis Age Chronic HCV infection AFP CHB infection Chronic hepatitis Alcohol consumption Alcohol history Sex ALT	- Variety of initial health statuses (diagnosed cirrhosis, CHB, carrier) - Prospective study - External validation in South Korea	- Ethnically homogenous cohort - Heavy alcohol use inconsistent variable - Limited antiviral available
CU-HCC [74]	Age Albumin Bilirubin HBV DNA Cirrhosis	- Treatment status heterogeneity - External validation [75] - Higher AUROC than REACH-B, NGM1-HCC, NGM2-HCC and GAG-HCC in North American population [76]	- Ethnically homogenous cohort - Did not discuss how missing data handled [77]
LSM-HCC [78]	Age Albumin HBV DNA LSM	- Further refined CU-HCC model - Treatment status heterogeneity - TE LSM more accurate than U/S [79,80] - Two-tier model risk stratification	- Ethnically homogenous cohort - Did not discuss how missing data handled [77]
GAG-HCC [81]	Version 1: Gender Age HBV DNA BCP mutations Cirrhosis Version 2: Gender Age HBV DNA Cirrhosis	- Continuous nature of some variables - 10-year NPV approaching 100% in cross validation [80] - Two-tier model for risk stratification	- Ethnically homogenous cohort - Same cohort for training and validation
REACH-B [82]	Gender Age ALT HBeAg HBV DNA	- Derived in Taiwan and applied to Hong Kong and South Korea - Large development cohort (n = 3584) - Used in APASL 2012 guidelines for anti-viral treatment eligibility [83] - Easy to evaluate and objective	- Limited discrimination in Caucasian population [84] - Not a validated predictor of anti-viral treatment eligibility in patients >40 - Three-tier stratification strategy - Developed in non-cirrhotic cohort - Only overt cirrhosis considered in exclusion criteria [82]
aMAP score [85]	Age Sex Albumin Bilirubin Platelet	- Generated, calibrated, and assessed in multiple ethnicities - Large derivation (3688) and validation (13,324) cohorts - Consistent performance with multiple etiologies/ethnicities - Continuous variables - Easy to evaluate and objective - Performed better than PAGE-B, LSM-HCC, REACH-B, CU-HCC, mREACH-B, mPAGE-B	- Platelet count as an indication of fibrosis [86] - PPV and specificity lower in external validation [87] - Three-tier stratification strategy - Intention to score for both HCV and HBV may limit HBV optimization
RWS-HCC [88]	Sex Age Cirrhosis AFP	- Minimal calculation required - Treatment status heterogeneity - Multiple Asian cohorts validation - Two-tier risk assessment strategy	- Ethnically homogenous cohort - Did not describe diagnosis of cirrhosis - Only patients with overt cirrhosis
NGM1-HCC [89]	Gender Age Family history of HCC Alcohol consumption ALT HBeAg	- Nomogram customizable for individual risk characteristics - Large HCC cohort (n = 3653) - Intuitive clinical decision tree	- Ethnically homogenous cohort - Alcohol consumption refers to frequency not volume of alcohol
NGM2-HCC [89]	Gender Age Family history of HCC Alcohol consumption ALT HBV DNA level	- Nomogram customizable for individual risk characteristics - Large HCC cohort (n = 3653) - Intuitive clinical decision tree	- Ethnically homogenous cohort - Alcohol consumption refers to frequency not volume of alcohol
LSPS [90]	Liver stiffness $\times \frac{Spleen\;diameter}{Platelet\;Count}$	- Potential multi-outcome predictor with precedence in esophageal varices, hepatic decompensation [91] - Population with heterogenous treatment status	- Ethnically homogenous cohort - Self-reported alcohol consumption was exclusion criteria - Three-tier stratification strategy - Intended for use as a marker
AGED [92]	Age Gender HBeAg HBV DNA	- Utility for non-cirrhotic CHB patient - Exclusively objective variables	- Ethnically homogenous cohort - Three-tier stratification system - Only overt cirrhosis was considered
D^2^AS Risk score [93]	HBV DNA Sex Age	- Evaluated multiple clinical indicators of cirrhosis - Continuous variables used - Common and objective variables	- Ethnically homogenous cohort - Minimal HBV genotype diversity - Did not evaluate well-known HCC risk factors, i.e., alcohol consumption

^1^ IPM: individual prediction model; CU-HCC: Chinese University HCC score; LSM-HCC: liver stiffness measurement-HCC; GAG-HCC: guide with age, gender, HBV DNA, core promoter mutations and cirrhosis; REACH-B: risk estimation for hepatocellular carcinoma in chronic hepatitis B; aMAP score: age, male, albumin-bilirubin, platelets; RWS-HCC: real world risk score for hepatocellular carcinoma; NGM1-HCC: nomogram 1 for HCC risk; NGM2-HCC: nomogram 2 for HCC Risk; LSPS: LS value-spleen diameter to platelet ratio score; AGED: age, gender, HBeAg and HBV DNA levels; D^2^AS: DNA^2^, age, sex.

**Table 3:** Critical Analysis of Treated Patient Risk Assessment Models.

Name ^1^	Components	Strengths	Weaknesses
PAGE-B [94]	Age Sex Platelets	- Validated in Asian populations [95,96] - Higher AUROC than REACH-B in Asian CHB patients [97] - Multiple categories for each variable - Common and objective variables	- Created using Caucasian dataset - Three-tier stratification system - Poorer AUROC than aMAP [85] - Development cohort exclusively patients receiving antiviral therapy
mREACH-B [98]	Gender Age ALT HBeAg LSM	- TE LSM more accurate than U/S [79,80] - Use of LSM values more likely to be accurate for treated patients - Validated in treatment heterogenous cohort [99]	- Retained majority of issues from REACH-B
AASL-HCC [100]	Age Albumin Sex Cirrhosis	- Comprehensive cirrhosis diagnosis - Higher accuracy for predicting 10-year risk of developing HCC than CU-HCC, GAG-HCC, REACH-B, Page-B	- Ethnically homogenous cohort - Three-tier stratification strategy - Homogenous HBV-C dominant genotype cohort
CAMD [101]	Cirrhosis Age Sex Diabetes	- Higher 5-year AUROC than mPAGE-B, PAGE-B in Korean population [102] - Different national cohorts for development and validation - Comprehensive cirrhosis diagnosis	- Ethnically homogenous cohort - Three-tier stratification strategy - Limited to clinically diabetic patients - No evidence for support in populations with high diabetes prevalence, i.e., South Asian
REAL-B [103]	Sex Age Alcohol Cirrhosis Diabetes Baseline Platelet Count Baseline AFP	- Study population included 8 different Asian ethnicities - One of the largest cohorts (n= 5356) - When compared to CAMD, had a better predictive value up to 10 years - Consistently capable in population with varying treatment type - In external validation, had greater discriminative performance than REACH-B, CU-HCC, GAG-HCC, PAGE-B and mPAGE-B [71]	- Limited to clinically diabetic patients - Limited to overt cirrhosis - Lack of explanation regarding quantity of “significant alcohol use” - Did not evaluate role of ethnicity or HBV genotype in accuracy
HCC-RESCUE [104]	Age Gender Cirrhosis	- Validated in Caucasian population using multiple antiviral therapies [105]	- Ethnically homogenous cohort - Three-tier stratification strategy - Limited to ultrasound for cirrhosis diagnosis
APA-B [106]	Age Platelet Count Baseline AFP	- Cohort with heterogenic cirrhosis profile - Confirmed and advised patients to avoid using herbal therapies concurrently - Easy to evaluate and objective	- Ethnically homogenous cohort - Three-tier stratification strategy - Did not account for impact of other variables on utility of platelet count as surrogate for cirrhosis

^1^ PAGE-B: platelets, age, gender-HBV score; mREACH-B: modified REACH-B; AASL-HCC: age, albumin, sex, liver cirrhosis)-HCC score; CAMD: cirrhosis, age, male sex, and diabetes mellitus score; REAL-B: real-world effectiveness from the Asia Pacific rim liver consortium for HBV; HCC-RESCUE: HCC-risk estimating score in CHB patients under entecavir; APA-B: age, platelet counts, and alpha-fetoprotein.
